# Surface Characteristics and Color Stability of Gingiva-Colored Resin Composites

**DOI:** 10.3390/ma13112540

**Published:** 2020-06-03

**Authors:** Aikaterini Petropoulou, Maria Dimitriadi, Spiros Zinelis, Aspasia Sarafianou, George Eliades

**Affiliations:** 1 Department of Prosthodontics, School of Dentistry, National and Kapodistrian University of Athens, 11527 Athens, Greece; aikatpetropoulou@gmail.com (A.P.); sarafia@otenet.gr (A.S.); 2 Department of Biomaterials, School of Dentistry, National and Kapodistrian University of Athens, 11527 Athens, Greece; mar.dimitriadi82@gmail.com (M.D.); szinelis@dent.uoa.gr (S.Z.)

**Keywords:** gingiva-colored composites, composition, degree of conversion, roughness, hardness, color stability

## Abstract

The purpose of this study was to investigate the surface characteristics and color stability of gingiva-colored composite restorative materials (Anaxgum—ANG, Ceramage—CMG and Gradia Gum—GRG). The microstructure, composition, degree of conversion (DC %) and 3D roughness (Sa, Sz, Sdr, Sc) were examined by LV-SEM/EDS, ATR-FTIR and optical profilometry, respectively. For the color stability (CIE L*, a*, b* system) and hardness (HV), measurements were performed at baseline and after 30 days storage in distilled water, coffee and red wine. The ANG and GRG contain prepolymerized particles in aromatic and aliphatic resin matrices, respectively, whereas CMG contains inorganic zirconia silicate/silica particles, in an aromatic resin matrix, with a smaller particle size and a higher surface area fraction. Urethane monomers were mainly identified in CMG and GRG. The DC% showed statistically insignificant differences between the materials. The same applied for the roughness parameters, except for the greatest Sdr in CMG. ANG showed a color difference (ΔE) of > 3.3 after immersion in all media, CMG in coffee and wine and GRG only in coffee. Sc was the only roughness parameter demonstrating correlations with the ΔL*, Δb* and ΔE*. The HV values showed insignificant differences between the storage conditions per material. There are important differences in the color stability of the materials tested, which were mostly affected by the roughness parameters due to variations in their microstructure.

## 1. Introduction

Successfully managing the pink aesthetics in implant prosthodontics is an important factor in cases of severe alveolar resorption, where the restoration of three-dimensional ridge deficiencies is required. Regenerative procedures have been used to solve this problem with varying degrees of success, since it is difficult to surgically reconstruct in a predictable way the most aesthetically critical areas, such as papillae and gingival margins [1,2].

Pink-colored ceramic materials have been used to satisfy the aesthetic demands in the anterior area. Nevertheless, achieving a match between ceramic and soft tissues is not always feasible, since a noticeable interface between the prosthetic and natural gingiva may exist [3]. Besides, the additional laboratory procedures required may create problems, such as the distortion of the framework and shrinkage during multiple ceramic firings, which may further compromise the final outcome [4]. Moreover, the repair of gingiva-colored ceramic, especially in cement-retained restorations, is a complicated procedure [3].

In an effort to overcome these problems, hybrid techniques have been developed. First, a customized screw-retained implant infrastructure has been proposed for soft tissue replacement with a light-cured gingiva-colored resin composite in combination with a cement-retained tooth superstructure [5,6]. Second, in screw-retained implant restorations, the teeth and gingival background are made of ceramic, whereas a final overlay of the pink contours is built intraorally with a direct gingiva-colored composite resin [3,7]. Finally, the metal framework of screw-retained restorations is exclusively veneered with indirect resin composites, tooth-colored for teeth and gingiva-colored for soft tissues [8,9].

Currently a significant number of direct and indirect gingiva-colored resin composites have been introduced. Indirect composites are more frequently used as a viable alternative to porcelain due to their improved mechanical properties, satisfactory aesthetics and handling characteristics in comparison with their direct analogues [3,10].

The aim of the present study was to evaluate the surface properties and color stability of some modern laboratory gingiva-colored composites. The null hypotheses were that (a) there are no differences in the surface properties, among the materials tested and (b) there are no differences in the color and hardness between and within the material groups after immersion in the staining media.

## 2. Materials and Methods

The materials tested are listed in Table 1.

### 2.1. Specimen Preparation

Disk-shaped thermoform molds (Ø = 8 mm, h = 2 mm) were placed on microscopic glass-slide surfaces covered with transparent cellulose matrix strips. The molds were filled with the composites, then covered with another set of strips and glass-slides and pressed to remove the material excess. Each specimen was irradiated for 40 s over the glass-slide using a halogen light-curing unit (Elipar Highlight, 3M ESPE, Seefeld, Germany, 900 mW/cm^2^ light intensity, standard mode) and then received a 5 min final curing in a laboratory polymerization unit (Labolight LV-III, GC Europe NV, 3 × 27 W fluorescent lamps). The specimens were stored at room temperature (23 ± 1 °C, 40% relative humidity, dark conditions) for 3 days, polished according to the procedures recommended by the manufacturers (Table 1), sonicated in water for 10 min to remove surface contaminants and dried in a desiccator. In all of the tests, the directly irradiated specimen surfaces were used for the measurements.

### 2.2. Surface Composition and Microstructure

The molecular composition of each product was examined by attenuated total internal reflection-Fourier transform infrared spectroscopy (ATR–FTIR). The unset materials (n = 3/product) were pressed against a single-reflection diamond element (Ø = 1 mm) of an ATR accessory (ZnSe lenses, 45° incidence angle, Golden-Gate MKII; Specac, Kent, UK) attached to an FTIR spectrometer (Spectrum GX; Perkin-Elmer, Buckinghamshire, UK), and the spectra were recorded under the following conditions: 4000–600 cm^−1^ range, 4 cm^−1^ resolution, 30 scans and ~1 μm sampling depth at 1000 cm^−1^.

The atomic number contrast backscattered electron images (BE) of the set and polished specimens (n = 3/product) were recorded to identify microstructural phase differences. The images were taken with a scanning electron microscope (SEM Quanta 200, FEI, Hilsboro, OR, USA) under the following conditions: Low vacuum mode (0.13 MPa chamber pressure), 20 kV accelerating voltage, 90 μA beam current and 500× magnification. Different mean atomic number phases were determined based on the gray-level discrimination and quantified by means of image analysis employing a dedicated software (XT Docu v3.2; Soft Imaging System GmbH, Münster, Germany). Each phase was quantified as the percentage coverage of the total specimen surface area imaged.

For the elemental composition, the same samples were examined by X-ray energy dispersive spectrometry (X-EDS), employing a spectrometer (Quantax, Bruker, Berlin, Germany) attached to the SEM equipped with a slew-window silicon drift detector (X Flash 6|10, Bruker) under low vacuum, 15 kV accelerating voltage, 108 μA beam current, area scan mode (250 μm × 220 μm), 200 s acquisition period and 34% dead time. The qualitative and quantitative analyses of the EDS spectra were performed in a non-standard mode employing atomic number, absorbance, fluorescence (ZAF) correction factors by the ESPRIT v.1.9 software (Bruker), with ~1 μm depth of analysis

### 2.3. Degree of Conversion

The degree of C=C bond conversion (DC%) of the set and polished specimens (n = 6/product) was examined by ATR-FTIR spectroscopy with the same methodology as described above. The directly irradiated surface of the set materials was pressed against the diamond ATR crystal by a sapphire anvil, and the spectra were recorded as before. The spectra of unset materials were used as controls. The DC% was calculated based on the two-band technique according to the equation:

DC% = 100 × [1 − (Ap_(C=C)_ × Am_(R)_/Am_(C=C)_ × Ap_(R)_)] (1)

where, A is the net peak absorbance height of the set (p) and unset (m) peaks of the methacrylate C=C bond stretching vibrations at 1636 cm^−1^, chosen as the analytical band and R the aromatic C..C stretching vibrations (1608 cm^−1^ for ANG, 1602 cm^−1^ for CMG) or urethane N–H bending vibrations (1545 cm^−1^ for GRG), used as reference bands, since these are not affected by the polymerization reaction.

### 2.4. Surface Roughness

The three-dimensional (3D) surface roughness of the set and polished specimens (n= 6/product) was evaluated by an optical profiler (Wyko NT 1100, Veeco, Tuscon, AZ, USA) operated in vertical scanning mode with a Mirau lens at a 40× magnification (113.3 μm × 148.5 μm sampling area) and a resolution of 0.1 nm (z-axis)/0.2 μm (x-, y-axes). Three measurements were performed on each specimen and averaged. The surface parameters evaluated were: Sa (average roughness), Sz (the average difference between the 5 highest peaks and 5 lowest valleys of consecutive sampling measurements), Sdr (percentage developed interfacial area ratio, the additional surface area contributed by the texture as compared to an ideal plane size of the measurement region) and Sc (core void volume, the volume supported by the surface from 10%–80% of the bearing ratio). All the parameters were determined using the Wyko Vision 32 software (Veeco).

### 2.5. Color Stability

Thirty-six set and polished specimens of each product were randomly divided into three groups (A-C, n = 12/product/group), individually annotated, stored in distilled water (72 h/37 °C) and blot-dried. The specimens were photographed under standard illumination, and then their CIE L*, a*, b* color coordinates were measured against a white background using a colorimeter (Dr Lange Microcolor Data Station, Braive Instruments, Liege, Belgium). Two measurements were recorded from each specimen with 90° sequential rotation and averaged. These values served as a reference. The specimens of each product were then immersed in 30 mL of coffee (group A, pH = 5), red wine (group B, pH = 3.4) and still water (group C, neutral pH) and stored in sealed opaque containers at 37 ± 1 °C. Every three days, the solutions were renewed and the specimens were rinsed with 30 mL of distilled water. After 30 days of storage, the specimens were rinsed with copious amounts of distilled water, blot-dried to remove visible moisture and photographed, and a second series of color measurements was performed. The color difference (ΔE, final-initial measurements) of each immersed specimen vs. its native control was calculated by the formula:

ΔE = [(ΔL*)^2^ + (Δa*)^2^ + (Δb*)^2^]^1/2^ (2)

### 2.6. Hardness

For each product, six specimens were randomly selected from the immersed groups (A–C). Hardness measurements were performed by a hardness tester (Diatronic 2RC, Wolpert, Ludwigshafen, Germany) equipped with a Vickers indenter under a 1 kp load, 70× magnification and 10 s contact period. On each specimen, three indentations were made in an equilateral triangular mode 2 mm distant from the margins, and the HV_1kp_ was averaged. A series of six additional set and polished specimens prepared per material as previously described and stored in distilled water (72 h/37 °C), served as a reference.

### 2.7. Statistical Analysis

For the DC% and roughness measurements which passed normality (Kolmogorov–Smirnov) and homoscedasticity (Levene’s) tests, a one-way ANOVA plus Tukey tests were used. For color stability, after normality testing a two-way ANOVA (using the type of material and aging solution as discriminating variables) plus Tukey tests were employed, whereas for hardness measurements that failed normality testing, a Κruscal–Wallis ANOVA on Ranks was executed and Tukey tests. Pearson’s correlation coefficient analysis was used to identify linear correlations between the surface roughness and color stability. The statistical analyses were performed with the Sigma Stat software (Sigma Plot v.12.5, Systat Software Inc, San Jose, CA, USA) at a 95% confidence level (α = 0.05).

## 3. Results

### 3.1. Composition and Microstructure

**Figure 1:**
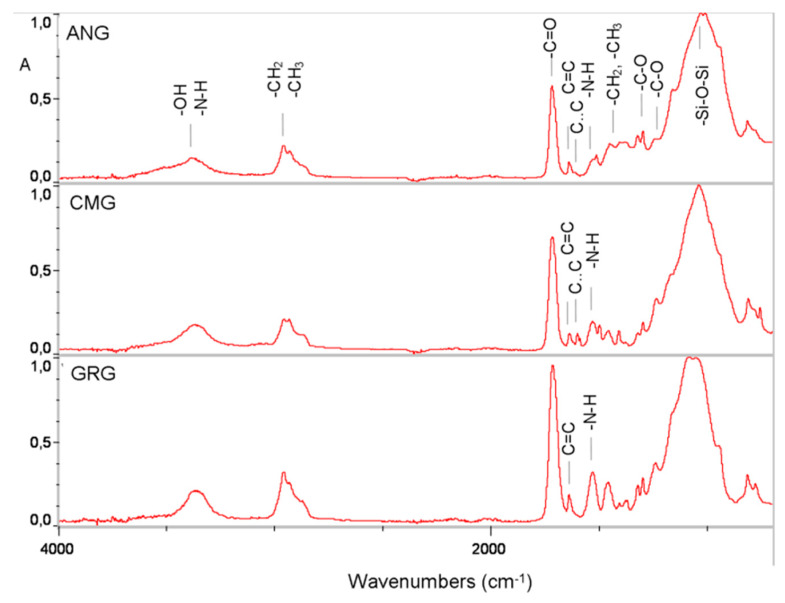
ATR-FTIR absorbance spectra of the unset materials.

The representative ATR-FTIR spectra of the unset materials are illustrated in Figure 1. The peak assignments are as follows (cm^−1^): O–H (3442, 1140–1110), N–H (3371), aromatic C..C (3010, 1608, 1595, 1510, 830, 801), CH_3_/CH_2_/CH (2920–2880, 1465–1430, 1370–1360, 720–700), C=O (1715, 1320, 1290), C=C (1634, 1500, 895), CON–H (1540), C–O–C (1260, 1105–1000) and Si–O (1150–1000) [11]. Based on this analysis, GRG is composed of aromatic-free methacrylates, including urethane compounds (UDMA); CMG contains aromatic and aliphatic methacrylates plus UDMA; and ANG contains aromatic and aliphatic methacrylates but with a small amount of UDMA, as shown by the low intensity of the CON–H vibrations (1540 cm^−1^) relative to the ester C=O vibrations (1715 cm^−1^).

**Figure 2:**
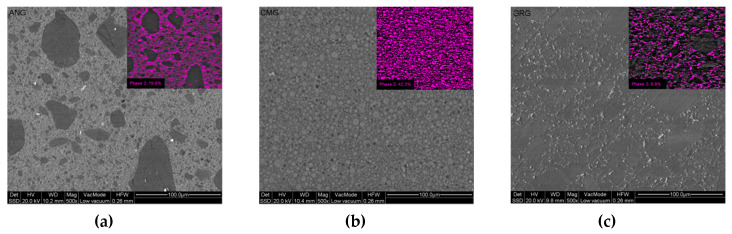
Atomic number contrast backscattered electron images of polished specimens of ANG (**a**), CMG (**b**) and GMG (**c**). The inserts demonstrate the area percentage of the colored phase (500×, bar = 100 μm).

The atomic number contrast backscattered electron images (BE) of the polished material surfaces are illustrated in Figure 2a–c. The microstructure of the ANG revealed the presence of low mean atomic number (ZL) particles ranging from 80 to 1 μm in size, which are prepolymerized resin composite particles. These particles, comprising approximately 80.2% of the surface area, were embedded in a resin matrix with fillers of a higher mean atomic number (ZH, 19.8% surface area). In CMG, two filler spherical particle phases were identified; one of ZL in low dispersion and a second of high ZH in high dispersion, the latter accounting for 42.3% of the surface area. For GRG, prepolymerized particles with maximum size of 7 μm were found, accounting for 93.1% of the surface area, embedded in a matrix with fillers of ZH (6.9% surface area).

**Figure 3:**
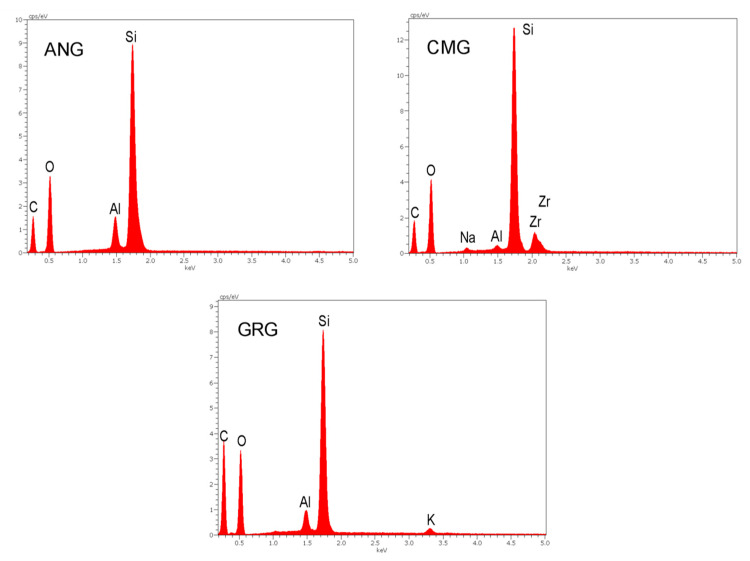
X-ray EDS spectra of the materials.

The X-ray EDS spectra of the polished material surfaces are presented in Figure 3. The results of the qualitative and quantitative analysis are summarized in Table 2.

The ANG and GRG showed a higher C and Al and a lower O and Si content than CMG. The latter demonstrated a significant amount of Zr (9.4 wt%).

### 3.2. Degree of Conversion

**Figure 4:**
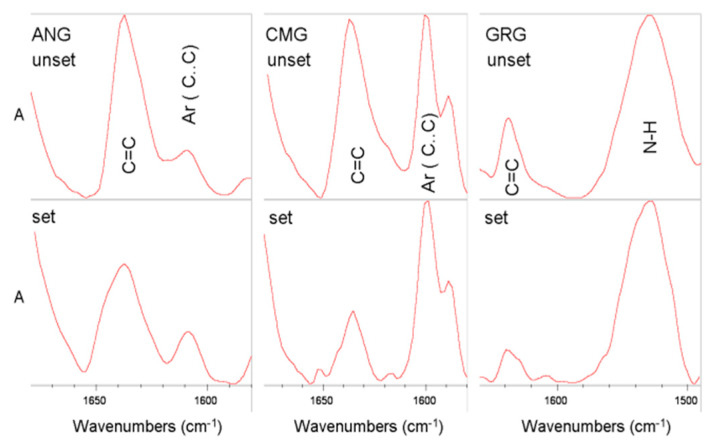
Expanded ATR-FTIR absorbance spectra used for the calculation of the degree of conversion (DC%) with the corresponding peaks.

The representative spectra used for the calculation of the DC%, demonstrating the analytical and reference bands per material, are illustrated in Figure 4. The results of the DC% (Table 3) ranged from 50.4% to 55%, with no statistically significant differences among the materials tested (*p* > 0.05).

### 3.3. Surface Roughness

**Figure 5:**
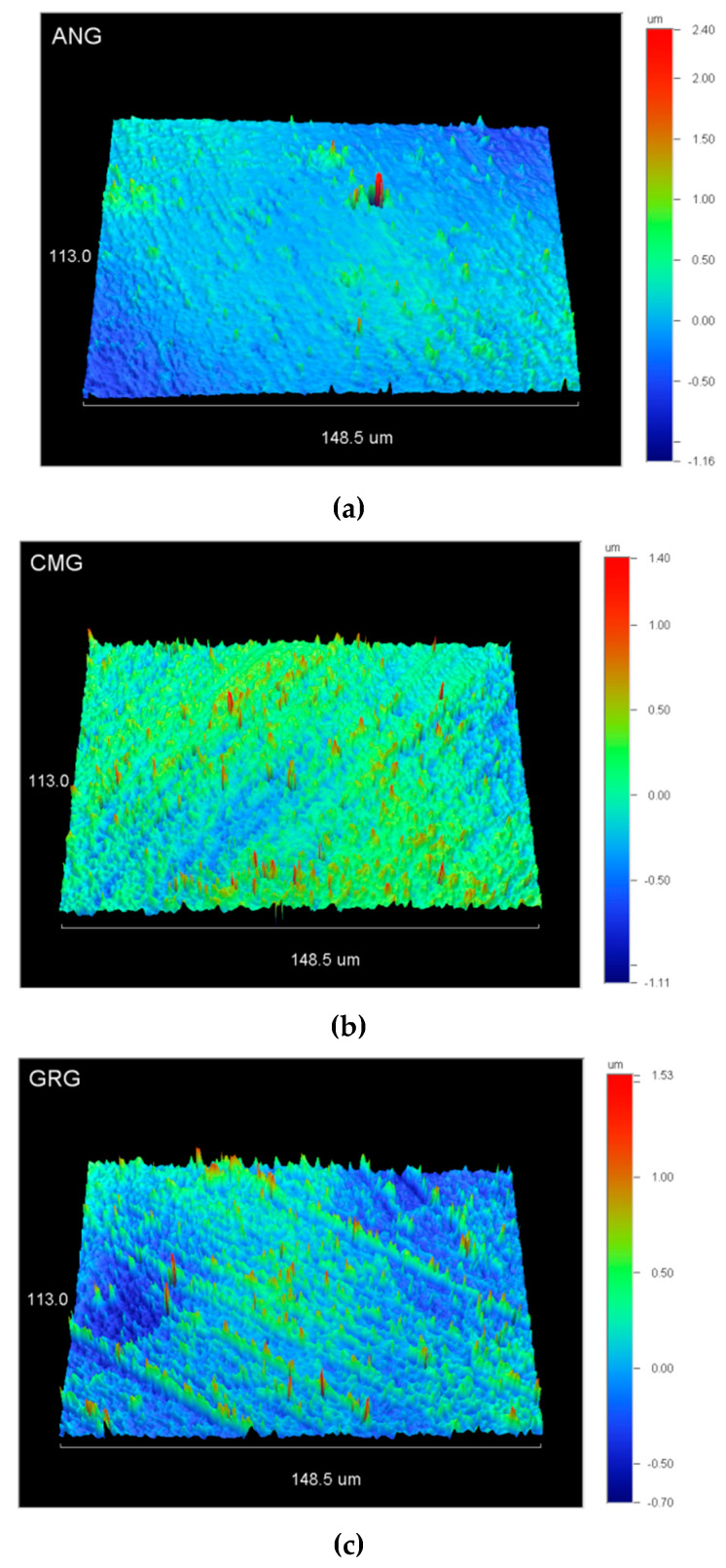
3D-profilometric images of (**a**) ANG (a, z-scale: −1.14~2.4 μm), (**b**) CMG (b, z-scale: −1.11~1.4 μm) and (**c**) GRG (c, z-scale: −0.7~1.53 μm) at 40× magnification.

The 3D profilometric images of the material surfaces after polishing with the methods advised by the manufacturers are illustrated in Figure 5. The results of the roughness parameters tested are summarized in Table 3. There were no statistically significant differences between the materials in the parameters tested except for CMG in Sdr, which demonstrated higher values than the ANG and CMG (*p* < 0.05).

### 3.4. Color Stability

**Figure 6:**
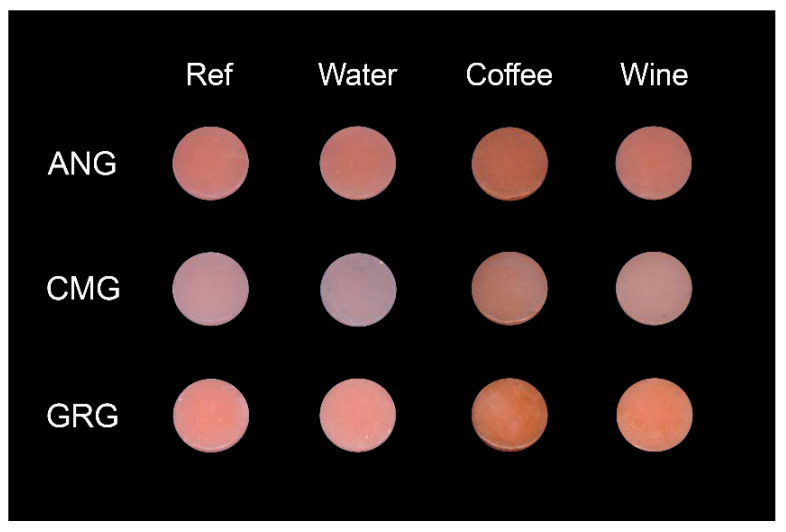
Photograph of the specimens before (ref) and after 30 days immersion in water, coffee and red wine.

Photographs of representative specimens before and after immersion in the testing solutions are presented in Figure 6. There were differences in the visual color appearance between the reference and the immersed materials. The reference materials ANG and GRG had a more pink color than the CMG. Water immersion did not affect the color of the ANG and GRG, but induced a blue-shift in the CMG. Coffee and wine had a major effect on the color of all the materials, creating a brown-shift, especially after coffee immersion, with CMG being the most discolored material. The quantitative results of the color parameters (L*, a*, b*) for the reference materials and the color differences (ΔL*, Δa*, Δb* and ΔE) after 30 d immersion in the testing solutions are presented in Table 4.

The effect of the storage medium was statistically significant for all the measurements, with a significant interaction between the storage medium and material type (*p* < 0.001). The latter was insignificant for ΔL* (*p* = 0.071) and Δa* (*p* = 0.292). For the reference materials, CMG demonstrated the highest values in L*, a*, and ANG the lowest in a* and b* (*p* < 0.05). The assessment of the effect of the immersion solutions on the same material revealed a reduction in ΔL* (CMG, GRG) after immersion in coffee; Δa* was mostly affected by water (ANG) and coffee (CMG, GRG), and Δb* by coffee in all the materials (*p* < 0.05). The ΔE values were the greatest after coffee immersion in all the materials, followed by wine in CMG (*p* < 0.05). Statistically insignificant ΔE differences were found in the ANG and GRG specimens immersed in water or wine (*p* > 0.05).

For the effect of the same solution on the different materials, water showed no effect on ΔL* (in all), significantly reduced Δa* in CMG and increased Δb* and ΔE in ANG and GMG. Coffee induced the most pronounced effects on CMG (ΔL*, Δa*, Δb*, ΔE; *p* < 0.05), with insignificant differences (*p* > 0.05) from GRG (Δa*) and ANG (Δb*). Finally, wine manifested the highest ΔL* in GRG, the lowest Δa* in ANG and the highest Δb* and ΔE in CMG (*p* < 0.05), with insignificant differences from GRG (ΔL*) and GMG (Δa*). For ANG, the ΔE values in all the immersion solutions presented visually perceptive color changes (ΔE > 3.3); for CMG, this only occurred in coffee and wine, and for GRG only in coffee. Coffee was the solution with the highest discoloration capacity for all the composites tested. A Pearson’s correlation coefficient analysis demonstrated significant correlations (coefficient values: 0.994) for ΔL* (wine-Sc, *p* = 0.0025), Δb* (wine-Sc, *p* = 0.0179) and ΔE (coffee-Sa, *p* = 0.039; coffee-Sc, *p* = 0.0097 and wine-Sz, *p* = 0.007).

### 3.5. Hardness

The results of the hardness measurements are summarized in Table 5. The storage conditions had no effect in the materials tested (*p* > 0.05). Under all storage conditions, the hardness (HV) values of CMG were the highest, the ANG values were ranked intermediate and the GRG values were the lowest.

## 4. Discussion

The results of the present study showed that there were no statistically significant differences among the materials tested in DC%, Sa, Sz, Sc, and HV_1kp_ per material before and after immersion; therefore, the null hypothesis for this part should be accepted. The significant HV_1kp_ differences between the materials per immersion medium render the hypothesis invalid, whereas for the complex color comparisons the hypothesis should be partially accepted, because many group differences were statistically significant.

The gingiva-colored composites used in the present study were mainly based on aromatic and aliphatic dimethacrylate monomers. Two contained aromatic monomers (ANG, CMG), as documented from the presence of aromatic C..C vibrations (1608, 1602, 1591 cm^−1^) in the ATR-FTIR spectra, and one contained only aliphatic monomers (GMG). In all the materials, CON-H peaks (1545 cm^−1^, not overlapping with the O-H peaks as at the 3450–3300 cm^−1^ band range) were identified, supporting the presence of UDMA monomers. Nevertheless, the intensity of this peak was the lowest of all in ANG, indicating a substantially reduced content. It is well documented that the type of monomer matrix affects a wide range of critical properties including color stability [12], with monomers possessing a more hydrophobic character being more stable. The choice of aliphatic UDMA monomer should be appended to the highest rate of polymerization, flexural modulus and strength vs. aromatic monomers (i.e., BisGMA, BisEMA) and the lower solubility than conventional aliphatic monomers, such as TEGDMA, which may balance and optimize resin matrix properties when used as a co-monomer [13,14]. The IR analysis of the resin matrix was incapable of identifying BDDMA, NPGDMA and TMPTMA, since their absorption bands were overlapping with those of all monomers. Nevertheless, the UDMA/BDDMA co-monomers have been shown to offer good color stability when combined with pyrogenic silica fillers [15]. The di-functional NPGDMA monomer forms highly branched polymers with very low shrinkage, despite its low molecular weight [16], increasing the crazing resistance of unfilled methacrylate polymers [17]. The tri-functional, low-viscosity, TMPTMA monomer demonstrates a high-crosslinking capacity, enhancing the cross-linking density of the polymers even during the auto-deceleration stage of polymerization [18]. This monomer, which has been successfully employed as a prepolymerized filler (20 μm size) in composite crowns for primary molars and in adhesive resin cements [19], has been advocated as a resin matrix co-monomer in resin composites replacing TEGDMA [20]. It is unclear if TMPTMA exists in GRG as a monomer or/and prepolymerized filler. Two of the materials tested were resin composites with prepolymerized particles (ANG, GRG) and one with purely inorganic particles (CMG). The particles reported for CMG were zirconium silicate. Based on the BE images of the polished surfaces, those particles with a size of <10 μm demonstrated a surface area of 42.3%. The atomic ratio of Zr/Si, as calculated from the wt% data of the EDS analysis (after dividing with the elemental atomic weight), was 0.11, indicating that an additional SiO_2_ filler fraction exists in ANG, since the theoretical Zr/Si atomic ratio in zirconia silicate is 1. This might be corroborated by the identification of the second lower atomic number phase found in low dispersion in ANG. In all products, Al was identified at <3.3 wt% content, with traces of Na and K possibly assigned to paste residues on polished material surfaces. The highest amount of Al found on ANG and GRG surfaces may indicate a greater impregnation capacity of alumina polishing paste due to the highest total resin content of these materials (resin matrix and prepolymerized resin-silica particles) or/and presence of Al containing glasses, as in GRG. The surface area of the prepolymerized particles in GMG (93.1%) was higher than that of ANG (42.3%), leading to a reduced prepolymer interparticle spacing in the former. ANG demonstrated a greater particle size distribution in the prepolymer particles than GMG, as shown in the BE images, but exhibited lower surface area (80.2%). These structural differences may affect the mechanical properties and surface quality of the restorations. The pink-colored resin composites usually contain FDA-approved red dyes to simulate gingiva color, such as 95% pure red iron oxide (FDA dye no 5595) or 85% disodium salt of 6-hydroxy 5(2-methyl-4-sulfophenyl) azol-2-naphthalene sulfonic acid (FDA dye no 40) at weight concentrations of 0.005%–0.75% relative to the resin weight component [21]. These concentrations are below the Fe and Na detection limits of the X-EDS analysis of the final product, as performed in the present study.

Despite the differences in monomer composition, there was no statistically significant difference in DC% between the materials tested. Therefore, the resin matrix conversion, which affects many material properties [22], does not seem to be a determinant factor for the properties tested in the present study. Possibly, the concentration of BisGMA, known to provide residual C=C unsaturation [23], was quite low to exert a significant effect, or the aliphatic co-monomers used were efficient in improving the overall conversion. The DC% values recorded were within the range previously reported for restorative composites [24] and marginal to the lower values offering an acceptable abrasive wear depth (~55%) for occlusal restorations [25]. Since gingiva-colored composites are not subjected to strong occlusal but rather to milder toothbrush abrasive wear, the DC values may be considered as acceptable.

Surface roughness, most often determined by the two-dimensional (2D) amplitude parameter Ra and more recently by the 3D-analogue Sa, is of paramount importance for plaque retention capacity [26,27] and the staining [28] of composite material surfaces. This is even more important for gingiva-colored composites because of their specific applications close to or in contact with gingival tissues. In the present study, the 3D roughness was assessed by including amplitude, hybrid and functional parameters for a more comprehensive characterization. The materials were hand-polished by a single operator utilizing the polishing media advised by each manufacturer. Therefore, although most clinically relevant, these values may not represent the ultimate polishing capacity of each material under standardized conditions, such as metallographic polishing [29]. The surface roughness of a resin composite is mainly determined by the composition, size, shape and quantity of the inorganic filler particles [30], with the smaller filler particles providing better finish [31]. The two materials with the prepolymerized resin-inorganic filler particles showed lower mean roughness parameters in all properties, apparently due to the submicron size of the inorganic particles in the prepolymer complexes. However, statistically significant differences were found only in Sdr. The observed high standard deviation values may imply the lack of standardization of the chair-side polishing procedures employing rotary instruments (brushes, points, etc.) on flat surfaces. For restorative resin composites polished with intraoral polishing systems Ra or Sa, values of 0.02~0.56 μm have been reported [24,28,29,30]. The materials tested in the present study after polishing with their individual systems demonstrated Sa values of 0.19~0.33 μm, which are within the previously reported range. It has been postulated that Ra values above 0.2 μm may result in increased plaque accumulation, leading to caries and periodontal disease risks [31]. Nevertheless, the rationale of making such correlations based on a single 2D amplitude roughness parameter from the great number of the currently available 3D parameters is put into question and requires further validation.

For the color stability of gingiva-colored composites after exposure to water and staining media, there is extremely limited information published. There is a single study by Khashayar, demonstrating the color instability of these composites even in tap water by employing the CIE L* a* b* system [32]. The three gingiva-colored composites showed significant color differences in the reference state; CMG was the lightest with the greatest red shift, whereas ANG was the only material demonstrating a blue color shift. This may be attributed to differences in the red pigment concentration. For all of the materials, coffee was the solution with the greatest staining capacity. The role of coffee as a strong staining agent has been already documented for tooth-colored composite restorative materials [33,34]. The results of the present study show that the same applies for pink-shaded gingiva-colored composites. The highest ΔE values were associated with high Δb* values, which is in agreement with previous findings [35]. Immersion in coffee induced the greatest yellow shift in CMG (15.4 absolute b* unit values), followed by GRG and ANG (10.6 absolute b* unit values), as coffee ingredients have a high affinity for organic polymer chains [36]. Although the ΔE differences correlated statistically with the Sa, Sc (coffee) and Sz (wine), there was no correlation of any of the roughness parameters tested with Δa*, which implies an effect independent of the surface roughness in the latter. For ΔL* and Δb*, the only correlations found were with Sc. The highest incidence of Sc correlations with the color co-ordinates tested was expected, as the latter expresses the volume retention capacity of a surface, which is more important for such applications from amplitude deviations (Sa, Sz) or extent of surface area ratio (Sdr). Red wine, combining the staining capacity of inherent dyes, such as tannins, with the plasticizing effect of the mild acidic pH [37,38], was ranked second in discoloration, again mainly affecting the Δb* values, especially in CMG (12.7 vs. 6.7–7 absolute b* unit values in ANG, GRG respectively). An interesting finding was that water storage induced a blue shift in all the materials, with the highest value recorded in ANG (−5.7 vs. 5.4 in CMG and 5.2 in GRG). It seems that in the absence of an external staining agent, water adsorption may change the mismatch in the refractive index between the resin matrix and the organic filler fraction of these materials, which, having a greater overall resin content absorb more water, showed an increased blue shift. However, surprisingly, this effect was visually more pronounced in CMG, which is filled with inorganic particles. The increased ΔL* in conjunction with the minimal changes in Δa* and Δb* after water storage may be a reasonable explanation. Nevertheless, the main contributing factor for the color changes in water seems to be the inherent instability of the red pigments used in these materials [32].

The results of the present study are in disagreement with previous studies, where it was reported that coffee produced less color change than red wine in tooth-colored resin composites [33,39], complying with the findings of Khashayar [32]. A possible explanation for this difference could be that the materials tested herein are gingiva-colored. Consequently, the red wine pigments did not alter the already red/pink color of the materials to the same extent as tooth-color materials, where the red color resolving capacity is much higher for profound reasons.

In the present study, a short-period static isothermal immersion test in staining media was performed without intermediate tooth-brushing. Although this design may have limited clinical relevance, it may facilitate the assessment of the early stages of stain absorption, without surface roughness interferences induced by tooth-brushing. Instead, intermediate water rinsing was routinely performed to remove the loosely bound fraction of the staining agents. The depth of staining associated with absorption or surface adsorption processes, the extent of color recovery after tooth-brushing or the need for re-finishing and re-polishing may provide important information for the clinical management of these materials and merit further investigation.

Hardness is an important property for composite materials, since it is implicated with their abrasion resistance. The highly filled inorganic particle-composite demonstrated a significantly higher hardness than the two prepolymerized particle-filled composites, as expected. Generally, the higher hardness is desirable from a clinical standpoint, as it provides increased scratch and wear resistance and thus an increased retention capacity of the original surface properties (i.e., roughness, gloss). Water storage for 30 days at 37 °C did not significantly affect the HV_1kp_ values of resin composites. This might be associated with the delayed post-curing reactions occurring in the warm bath, which may hinder the expression of the plasticizing effect of the inert and neutral solvent [40]. Similar observations apply for the effect of the weak and mild acidic coffee and wine solutions, respectively. Nevertheless, further storage may induce plasticization, especially under low pH conditions, which promotes the acidic degradation of the polyester matrix, organic fillers and the silane coupling agent at the inorganic filler-matrix interface [41].

## 5. Conclusions

Summarizing the results of the present study and considering the limitations of the methodology employed, the following conclusions can be drawn:

- The gingiva-colored resin composites were susceptible to staining, with coffee producing the greatest color changes in all the materials tested.
- There was no statistically significant difference in the degree of conversion between the materials. Hardness was not affected by the immersion media. Nevertheless, the hardest inorganic particle-filled material demonstrated the highest Sdr values and ΔE changes in coffee and wine.
- From the surface roughness parameters tested, only the functional parameter Sc, expressing the volume retention capacity of a surface, showed correlations with ΔL*, Δb* and ΔE.

## Figures and Tables

**Table 1:** The gingiva-colored resin composites used in the study.

Material (Code)	Composition*	Finishing/Polishing Procedure*	Manufacturer
AnaxGUM (ANG) Shade: Light Pink	Resin: UDMA, BDDMA, BisGMA Filler: Glass, pyrogenic SiO_2_ (74 wt%, 0.7 μm)	Polishing brush and Pasta Grigia II	Anaxdent GmbH Stuttgard, Germany
Ceramage Body (CMG) Shade: Gum-L	Resin: UDMA, dimethacrylates Filler: ZrSiO_4_ (73 wt% progressed fine structure filler)	CompoMaster (diamond impregnated silicone points coarse, hight-lustre) Dura-Polish, Dura-Polish DIA (alumina pastes)	Shofu Kyoto, Japan
Gradia Gum (GRG) Shade: G23	Resin: UDMA, NGDMA, TMPTMA Filler: Trimodal (pre-polymerized particles, AlBSiO_4_, SiO_2_, 75 wt%)	CompoMaster (diamond impregnated silicone points coarse, hight-lustre) Gradia Diapolisher paste	GC Europe NV Leuven, Belgium

* According to manufacturers’ information. UDMA: Urethane dimethacrylate; BDDMA: Butanodiol dimethacrylate; BisGMA: Bisphenol glycidyl dimethacrylate; NGDMA: Neopentyl glycol dimethacrylate; TMPTMA: Trimethylol propane trimethacrylate.

**Table 2:** The results of the elemental analysis of the materials tested (wt%, means of 3 measurements).

Material	Elemental Composition (wt%)						
	C	O	Si	Al	Zr	Na	K
**ANG**	55.4	21.5	19.8	3.3	-	-	-
**CMG**	31.9	31.7	25.6	0.7	9.4	0.7	-
**GRG**	55.7	22.9	18.2	2.2	-	-	0.9

**Table 3:** The results of the DC% and surface roughness parameters (means and standard deviations).

Material	DC%	Sa (nm)	Sz (μm)	Sdr (%)	Sc (nm^3^/nm^2^)
**ANG**	55 (2.6)^a^	221.5 (131.6)^a^	2.1 (1.2)^a^	3.7 (1.3)^a^	310.4 (174.7)^a^
**CMG**	54.6 (2.9)^a^	306.4 (177.9)^a^	3.2 (1.6)^a^	9.2 (3.6)^b^	388.8 (203.4)^a^
**GRG**	50.4 (5.9)^a^	188.3 (49)^a^	2.0 (0.2)^a^	4.3 (1.7)^a^	267.7 (99.4)^a^

Same superscript letters show values with statistically insignificant differences per property (*p* > 0.05).

**Table 4:** The results of the color parameters (means and standard deviations).

GROUPS	ANG				CMG				GRG			
	L*	a*	b*		L*	a*	b*		L*	a*	b*	
**Reference**	50.58 (0.78) ^A^	13.91 (0.46) ^A^	−3.19 (0.59) ^A^		57.33 (1.56) ^B^	23.21 (0.84) ^B^	5.78 (0.22) ^B^		50.01 (0.9) ^A^	21.2 (0.57) ^C^	5.58 (0.28) ^B^	50.58 (0.78) ^A^
**After 30d immersion**	ΔL*	Δa*	Δb*	ΔE*	ΔL*	Δa*	Δb*	ΔE*	ΔL*	Δa*	Δb*	ΔE*
Water	0.73 (0.87) ^a,A^	−2.83 (1.06) ^a,A^	−2.50 (0.82) ^a,A^	3.99 (1.16) ^a,A^	1.24 (1.50) ^a,A^	0.07 (0.78) ^a,B^	−0.36 (0.27) ^a,B^	1.79 (1.12) ^a,B^	0.33 (0.91) ^a,A^	−1.13 (0.90) ^a,C^	−0.37 (0.34) ^a,B^	1.63 (0.72) ^a,B^
Coffee	1.49 (1.68) ^a,A^	−1.30 (1.17) ^a,b,A^	7.41 (1.30) ^b,A,B^	7.88 (1.58) ^b,A^	−4.18 (1.97) ^b,B^	−3.69 (1.39) ^b,B,C^	9.64 (2.06) ^b,B^	11.37 (2.12) ^b,B^	−0.53 (0.99) ^b,A^	−3.09 (1.15) ^b,B,^	5.05 (0.94) ^b,A^	6.09 (1.11) ^b,C^
Wine	0.93 (1.48) ^a,A,B^	−0.72 (0.60) ^b,A^	3.50 (0.32) ^c,A^	3.99 (0.34) ^a,A^	0.08 (1.64) ^a,A^	−2.08 (1.03) ^b,B^	6.93 (1.30) ^b,B^	7.51 (1.04) ^c,B^	1.40 (0.78) ^a,B^	−1.38 (1.15) ^a,b,A,B^	1.42 (0.30) ^c,A^	2.67 (0.79) ^a,C^

For reference groups, the same upper-case letters show statistically insignificant differences (*p* > 0.05) between materials per parameter (L*, a*, b*). For the immersed specimens, upper-case letters demonstrate insignificant differences in color difference (ΔE) and the corresponding parameters (final-reference values), whereas lower-case letters denote insignificant differences per material and parameter between the immersion media.

**Table 5:** Results of the hardness measurements (HV_1kp_ units) of the materials tested following immersion in water and the staining media (median and 25%–75% percentiles).

GROUPS	ANG	CMG	GRG
Reference	50.3 (46.6–54) ^a,A^	75.1 (68.9–75.3) ^a,B^	37 (35.8–38.1) ^a,C^
Water	56.2 (54–58.4) ^a,A^	75.3 (75.3–82.5) ^a,B^	38.2 (33.6–40.7) ^a,C^
Coffee	48.4 (46.6–50.1) ^a,A^	80.1 (60.9–84.9) ^a,B^	40.7 (40.7–43.5) ^a,C^
Wine	45.4 (40.7–58.4) ^a,A^	70.1 (66.3–74.9) ^a,B^	38.1 (38.1–40.7) ^a,C^

Same lower-case superscript letters show median values with no statistically significant differences per material and upper-case between materials per treatment (*p* > 0.05).

## References

[1] Coachman C., Salama M., Garber D., Calamita M., Salama H., Cabral G. (2009). Prosthetic Gingival Reconstruction in a Fixed Partial Restoration. Part 1: Introduction to Artificial Gingiva as an Alternative Therapy. Int. J. Periodontics Restor. Dent..

[2] Thoma D.S., Mόhlemann S., Jung R.E. (2014). Critical Soft-Tissue Dimensions with Dental Implants and Treatment Concepts. Periodontology 2000.

[3] Coachman C., Salama M., Garber D., Calamita M., Salama H., Cabral G. (2010). Prosthetic Gingival Reconstruction in Fixed Partial Restorations. Part 3: Laboratory Procedures and Maintenance. Int. J. Periodontics Restor. Dent..

[4] Mackert J.R., Williams A.L. (1996). Microcracks in Dental Porcelain and Their Behavior during Multiple Firing. J. Dent. Res..

[5] Hagiwara Y., Nakajima K., Tsuge T., McGlumphy E.A. (2007). The Use of Customized Implant Frameworks with Gingiva-Colored Composite Resin to Restore Deficient Gingival Architecture. J. Prosthet. Dent..

[6] Malσ P., de Araϊjo Nobre M., Borges J., Almeida R. (2012). Retrievable Metal Ceramic Implant-Supported Fixed Prostheses with Milled Titanium Frameworks and All-Ceramic Crowns: Retrospective Clinical Study with up to 10 Years of Follow-Up. J. Prosthodont. Implant Esthet. Reconstr. Dent..

[7] Petropoulou A., Pappa E., Pelekanos S. (2013). Esthetic Considerations When Replacing Missing Maxillary Incisors with Implants: A Clinical Report. J. Prosthet. Dent..

[8] Geckili O., Bilhan H., Ceylan G., Cilingir A. (2013). Edentulous Maxillary Arch Fixed Implant Rehabilitation Using a Hybrid Prosthesis Made of Micro-Ceramic-Composite: Case Report. J. Oral Implant..

[9] Petropoulou A., Pantzari F., Nomikos N., Chronopoulos V., Kourtis S. (2013). The Use of Indirect Resin Composites in Clinical Practice: A Case Series. Dentistry.

[10] An H.-S., Park J.-M., Park E.-J. (2011). Evaluation of Shear Bond Strengths of Gingiva-Colored Composite Resin to Porcelain, Metal and Zirconia Substrates. J. Adv. Prosthodont..

[11] Williams B., Braden M. (1981). Characteristics of fissure sealants. J. Dent. Res..

[12] Fonseca A.S.Q.S., Labruna Moreira A.D., de Albuquerque P.P.A.C., de Menezes L.R., Pfeifer C.S., Schneider L.F.J. (2017). Effect of Monomer Type on the CC Degree of Conversion, Water Sorption and Solubility, and Color Stability of Model Dental Composites. Dent. Mater..

[13] Sideridou I., Tserki V., Papanastasiou G. (2003). Study of Water Sorption, Solubility and Modulus of Elasticity of Light-Cured Dimethacrylate-Based Dental Resins. Biomaterials.

[14] Gajewski V.E.S., Pfeifer C.S., Frσes-Salgado N.R.G., Boaro L.C.C., Braga R.R. (2012). Monomers Used in Resin Composites: Degree of Conversion, Mechanical Properties and Water Sorption/Solubility. Braz. Dent. J..

[15] Ren Y.-F., Feng L., Serban D., Malmstrom H.S. (2012). Effects of Common Beverage Colorants on Color Stability of Dental Composite Resins: The Utility of a Thermocycling Stain Challenge Model in Vitro. J. Dent..

[16] Cowperthwaite G.F., Foy J.J., Malloy M.A., Gebelein C.G., Koblitz F.F. (1981). The Nature of the Crosslinking Matrix Found in Dental Composite Filling Materials and Sealants. Biomedical and Dental Applications of Polymers.

[17] Burchill P.J., Mathys G., Stacewicz R.H. (1987). Analysis and Properties of Some Commercial Poly(Methylmethacrylate)-Based Materials. J. Mater. Sci..

[18] Stansbury J.W. (2012). Dimethacrylate Network Formation and Polymer Property Evolution as Determined by the Selection of Monomers and Curing Conditions. Dent. Mater..

[19] Wada K., Ikeda E., Wada J., Inoue G., Miyasaka M., Miyashin M. (2016). Wear Characteristics of Trimethylolpropane Trimethacrylate Filler-Containing Resins for the Full Crown Restoration of Primary Molars. Dent. Mater. J..

[20] da Silva E.M., Miragaya L., Noronha-Filho J.D., Amaral C.M., Poskus L.T., Guimarγes J.G.A. (2016). Characterization of an Experimental Resin Composite Organic Matrix Based on a Tri-Functional Methacrylate Monomer. Dent. Mater. J..

[21] Barnes N., Feinman R., Waknine S., Alpert B.H. (1995). Gum-Colored Dental Composite and Dental Restoration Kit. US Patent.

[22] Ruyter I.E. (1982). Methacrylate-Based Polymeric Dental Materials: Conversion and Related Properties. Summary and Review. Acta Odontol. Scand..

[23] Pereira S.G., Nunes T.G., Kalachandra S. (2002). Low Viscosity Dimethacrylate Comonomer Compositions [Bis-GMA and CH3Bis-GMA] for Novel Dental Composites; Analysis of the Network by Stray-Field MRI, Solid-State NMR and DSC & FTIR. Biomaterials.

[24] Silikas N., Kavvadia K., Eliades G., Watts D. (2005). Surface Characterization of Modern Resin Composites: A Multitechnique Approach. Am. J. Dent..

[25] Ferracane J.L., Mitchem J.C., Condon J.R., Todd R. (1997). Wear and Marginal Breakdown of Composites with Various Degrees of Cure. J. Dent. Res..

[26] Bollen C.M., Lambrechts P., Quirynen M. (1997). Comparison of Surface Roughness of Oral Hard Materials to the Threshold Surface Roughness for Bacterial Plaque Retention: A Review of the Literature. Dent. Mater..

[27] Teranaka A., Tomiyama K., Ohashi K., Miyake K., Shimizu T., Hamada N., Mukai Y., Hirayama S., Nihei T. (2018). Relevance of Surface Characteristics in the Adhesiveness of Polymicrobial Biofilms to Crown Restoration Materials. J. Oral Sci..

[28] Lu H., Roeder L.B., Lei L., Powers J.M. (2005). Effect of Surface Roughness on Stain Resistance of Dental Resin Composites. J. Esthet. Restor. Dent..

[29] Ereifej N.S., Oweis Y.G., Eliades G. (2013). The Effect of Polishing Technique on 3-D Surface Roughness and Gloss of Dental Restorative Resin Composites. Oper. Dent..

[30] Ruivo M.A., Pacheco R.R., Sebold M., Giannini M. (2019). Surface Roughness and Filler Particles Characterization of Resin-Based Composites. Microsc. Res. Tech..

[31] Heintze S.D., Forjanic M., Ohmiti K., Rousson V. (2010). Surface Deterioration of Dental Materials after Simulated Toothbrushing in Relation to Brushing Time and Load. Dent. Mater..

[32] Khashayar G. (2013). Color Science in Dentistry. Ph.D. Thesis.

[33] Kentrou C., Papadopoulos T., Lagouvardos P. (2014). Color Changes in Staining Solutions of Four Light-Cured Indirect Resin Composites. Odontology.

[34] Poggio C., Ceci M., Beltrami R., Mirando M., Wassim J., Colombo M. (2016). Color Stability of Esthetic Restorative Materials: A Spectrophotometric Analysis. Acta Biomater. Odontol. Scand..

[35] Seghi R.R., Gritz M.D., Kim J. (1990). Colorimetric Changes in Composites Resulting from Visible-Light-Initiated Polymerization. Dent. Mater..

[36] Park J.-K., Kim T.-H., Ko C.-C., Garcνa-Godoy F., Kim H.-I., Kwon Y.H. (2010). Effect of Staining Solutions on Discoloration of Resin Nanocomposites. Am. J. Dent..

[37] Asmussen E., Peutzfeldt A. (2001). Influence of Pulse-Delay Curing on Softening of Polymer Structures. J. Dent. Res..

[38] Ardu S., Braut V., Gutemberg D., Krejci I., Dietschi D., Feilzer A.J. (2010). A Long-Term Laboratory Test on Staining Susceptibility of Esthetic Composite Resin Materials. Quintessence Int. Berl. Ger. 1985.

[39] Anfe T.E.d.A., Agra C.M., Vieira G.F. (2011). Evaluation of the Possibility of Removing Staining by Repolishing Composite Resins Submitted to Artificial Aging. J. Esthet. Restor. Dent..

[40] Milleding P., Ahlgren F., Wennerberg A., Ortengren U., Karlsson S. (1998). Microhardness and Surface Topography of a Composite Resin Cement after Water Storage. Int. J. Prosthodont..

[41] Sφderholm K.-J., Zigan M., Ragan M., Fischlschweiger W., Bergman M. (2016). Hydrolytic Degradation of Dental Composites. J. Dent. Res..

